# Safety and efficacy of low-dose sirolimus in the PIK3CA-related overgrowth spectrum

**DOI:** 10.1038/s41436-018-0297-9

**Published:** 2018-10-01

**Authors:** Victoria E. R. Parker, Kim M. Keppler-Noreuil, Laurence Faivre, Maxime Luu, Neal L. Oden, Leena De Silva, Julie C. Sapp, Katrina Andrews, Marc Bardou, Kong Y. Chen, Thomas N. Darling, Elodie Gautier, Barry R. Goldspiel, Smail Hadj-Rabia, Julie Harris, Georgios Kounidas, Parag Kumar, Marjorie J. Lindhurst, Romaric Loffroy, Ludovic Martin, Alice Phan, Kristina I. Rother, Brigitte C. Widemann, Pamela L. Wolters, Christine Coubes, Lucile Pinson, Marjolaine Willems, Catherine Vincent-Delorme, PROMISE Working Group, Pierre Vabres, Robert K. Semple, Leslie G. Biesecker

**Affiliations:** 1 Institute of Metabolic Science, University of Cambridge, Cambridge, UK; 2 Medical Genomics and Metabolic Genetics Branch, National Human Genome Research Institute, National Institutes of Health, Bethesda, MD USA; 3 Centres de références Anomalies du Développement et Anomalies Dermatologiques Rares, Equipe GAD UMR1231 et FHU TRANSLAD, CHU Dijon-Bourgogne et Université de Bourgogne, Dijon, France; 4 Centre d’Investigation Clinique INSERM 1432, Centre Hospitalier Universitaire de Dijon, Dijon, Bourgogne France; 5 The EMMES Corporation, Rockville, MD USA; 6 Section on Pediatric Diabetes and Metabolism, National Institute of Diabetes, Digestive, and Kidney Diseases, National Institutes of Health, Bethesda, MD USA; 7 Department of Dermatology, Uniformed Services University of the Health Sciences, Bethesda, MD USA; 8 Pharmacy Department, NIH Clinical Center, National Institutes of Health, Bethesda, MD USA; 9 Department of Dermatology and Reference Center for Genodermatoses and Rare Skin Diseases (MAGEC), Université Paris Descartes - Sorbonne Paris Cité, INSERM U1163, Institut Imagine, Institut Imagine, Hôpital Universitaire Necker-Enfants Malades, Paris, France; 10 Department of Interventional Radiology, Dijon University Hospital, Dijon, France; 11 Department of Dermatology, University Hospital Center of Angers, Angers, France; 12 Department of Dermatology, Claude Bernard-Lyon 1 University and Hospices Civils de Lyon, Lyon, France; 13 NCI, CCR, Pediatric Oncology Branch, National Institutes of Health, Bethesda, MD USA; 14 Département de Génétique Médicale, Maladies Rares et Médecine Personnalisée, CHU de Montpellier, Montpellier, France; 15 Service de Génétique médicale, Hôpital Jeanne de Flandre, CHRU de Lille, Lille, France; 16 Centre for Cardiovascular Science, Queen’s Medical Research Institute, University of Edinburgh, Edinburgh, UK

**Keywords:** overgrowth, mosaicism, *PIK3CA*, sirolimus

## Abstract

**Purpose:**

*PIK3CA*-related overgrowth spectrum (PROS) encompasses a range of debilitating conditions defined by asymmetric overgrowth caused by mosaic activating *PIK3CA* variants. *PIK3CA* encodes the p110α catalytic subunit of phosphatidylinositol-3-kinase (PI3K), a critical transducer of growth factor signaling. As mTOR mediates the growth-promoting actions of PI3K, we hypothesized that the mTOR inhibitor sirolimus would slow pathological overgrowth.

**Methods:**

Thirty-nine participants with PROS and progressive overgrowth were enrolled into open-label studies across three centers, and results were pooled. For the primary outcome, tissue volumes at affected and unaffected sites were measured by dual energy X-ray absorptiometry during 26 weeks of untreated run-in and 26 weeks of sirolimus therapy.

**Results:**

Thirty participants completed the study. Sirolimus led to a change in mean percentage total tissue volume of –7.2% (SD 16.0, *p* = 0.04) at affected sites, but not at unaffected sites (+1.7%, SD 11.5, *p* = 0.48) (*n* = 23 evaluable). Twenty-eight of 39 (72%) participants had ≥1 adverse event related to sirolimus of which 37% were grade 3 or 4 in severity and 7/39 (18%) participants were withdrawn consequently.

**Conclusion:**

This study suggests that low-dose sirolimus can modestly reduce overgrowth, but cautions that the side-effect profile is significant, mandating individualized risk–benefit evaluations for sirolimus treatment in PROS.

## Introduction

*PIK3CA*-related overgrowth spectrum (PROS) designates a heterogeneous group of rare, asymmetric overgrowth disorders caused by postzygotic variants in the gene *PIK3CA*.^1^ *PIK3CA* encodes the p110α catalytic subunit of phosphoinositide 3-kinase (PI3K), which transduces activation of tyrosine kinase growth factor and hormone receptors into activation of AKT and mTOR signaling^2^ to promote tissue growth. *PIK3CA* variants in PROS cause physiologically inappropriate activation of AKT and mTOR, and variable, asymmetric overgrowth, consistent with causation by an early developmental stochastic pathogenic variant. Overgrowth includes adipose tissue, muscle, skin, bone, blood or lymph vessels, or neural tissue, among others.^3–6^ Adipose and vascular components are particularly striking, reflecting the inherent plasticity and postnatal growth potential of these tissues. Complications of PROS depend on the anatomical site and extent of overgrowth, but may include functional impairment (e.g., of walking or swallowing), pain, recurrent superficial infections, thromboembolism, and/or hemorrhage, all of which may be debilitating, and cause early mortality. Current treatment is inadequate, relying on debulking surgery, amputation, and/or endovascular occlusive procedures. Regrowth following surgery occurs frequently and repeated surgery is common.^7^

Some genotype–phenotype correlation in PROS has been suggested,^8,9^ however the dominant determinant of phenotype is the timing and location of the pathogenic variant . This causes a high degree of interindividual phenotypic heterogeneity in PROS. Compounding this, growth trajectories vary greatly for unknown reasons, with some exhibiting excess growth limited to childhood, while others have progressive soft tissue overgrowth during adult life. Finally, overgrown tissue usually lacks the clear anatomical demarcation characteristic of neoplasia on imaging. Collectively these considerations complicate the design of quantitative endpoints for therapeutic studies.

Allosteric mTOR inhibitors such as sirolimus are approved for posttransplant immunosuppression. They can also slow the growth of cancers bearing *PIK3CA* variants.^10^ Sirolimus potently attenuates pathological AKT signaling and reduces cell proliferation in dermal fibroblasts derived from people with PROS,^11,12^ which suggests that it could be an effective treatment of PROS. Anecdotal reports of sirolimus therapy in PROS have suggested efficacy,^13,14^ while other studies have reported clinical improvement in patients with vascular overgrowth caused by *TIE2* variants^15^ or of unknown cause.^16,17^ Further, unpublished patient and clinician reports suggest wider off-label use of sirolimus in PROS. To assess the potential efficacy of sirolimus in PROS further, and to address the challenges of study design, we undertook a study of low-dose sirolimus in patients with molecularly proven PROS, assessing quantitative disease endpoints. The study was conducted across three centers using a common protocol.

## Materials and methods

This pilot study was performed in accordance with the Declaration of Helsinki and Good Clinical Practice guidelines or the US human subject research regulations (US Code of regulations 45CFR46). The study protocol was approved by ethics review boards of each study site. Regulatory approval from the Medicines and Healthcare Products Regulatory Agency (MHRA) was obtained in the United Kingdom, and Agence Nationale de Sécurité du Médicament et des Produits de Santé (ANSM) in France. Written informed consent and/or parental consent and where possible assent for the participation of children was obtained for all participants. Study Registration: France (NCT02443818), United Kingdom (EudraCT: 2014-000484-41), United States (NCT02428296).

### Study overview and design

**Fig. 1:**
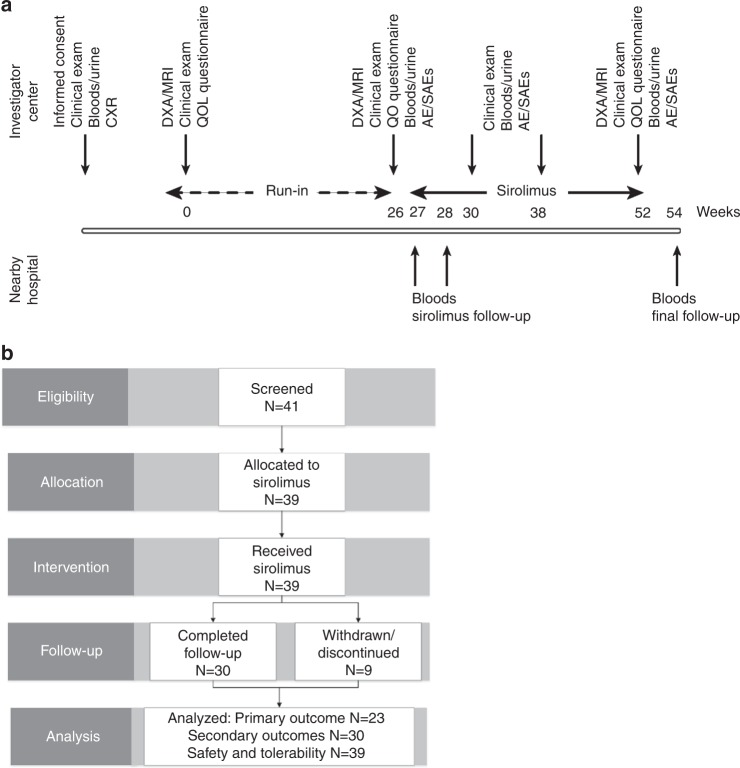
**CONSORT flow-chart and schematic of nonrandomized open-label pilot study.** **a** Schematic of number of participants assessed for eligibility and excluded or allocated to the study, treated, followed, and analyzed. Of the 39 subjects enrolled, 30 completed 26 weeks of sirolimus therapy, and 23/30 had anatomy that permitted analysis of the primary outcome measure. Safety and tolerability were evaluated in all treated participants. **b** Overview of study design including schedule of procedures. *AE/SAEs* adverse events/serious adverse events, *CXR* chest X-ray, *DXA/MRI* dual energy X-ray absorptiometry/magnetic resonance imaging, *QOL* quality of life.

We selected a nonrandomized, open-label design with a 26-week observational run-in period to measure growth (Fig. 1a). The study was conducted at Cambridge University Hospitals NHS Trust (United Kingdom), CHU Dijon-Bourgogne (France), and the National Human Genome Research Institute (United States) (Supplemental Table S1). Intraparticipant comparisons between affected and matched unaffected contralateral tissue were made where possible. A placebo was excluded pragmatically due to growing awareness of the availability of sirolimus and its increasing off-license use in PROS, potentially compromising recruitment within this orphan disease population. Participants were assessed for sirolimus efficacy at week 0 (before the run-in phase), week 26 (after the run-in phase and before treatment), and at week 52 (after 26 weeks of treatment).

### Inclusion/exclusion and withdrawal criteria

Eligible participants were aged from 3 to 65 years, inclusive. All had a diagnosis of *PIK3CA*-related overgrowth spectrum (PROS)^9,18^ with progressive overgrowth (determined by self-report or serial measurements) and a mosaic *PIK3CA* variant identified by methodology described previously.^5,9,18^ (Table 1).

**Table 1:** Baseline characteristics of enrolled participants

	UK	France	US	Combined
	*N* = 11	*N* = 15	*N* = 13	*N* = 39
**Mean age, years (range)**	19.8 (5–44)	16.4 (3–39)	13.6 (3–48)	16.6 (3–48)
**Gender F:M**	4:7	7:8	6:7	17:22
***PIK3CA*** **variant**				
c.3132T>A p.(Asn1044Lys)			1/13 (8%)	1/39 (3%)
c.31327A>G p.(Met1043Val)		1/15 (7%)		1/39 (3%)
c.31340A>G p.(His1047Arg)	4/11 (36%)	3/15 (20%)	4/13 (31%)	11/39 (28%)
c.3140A>T p.(His1047Leu)		2/15 (13%)	1/13 (8%)	3/39 (8%)
c.3139C>T p.(His1047Tyr)			1/13 (8%)	1/39 (3%)
c.3073A>G p.(Thr1025Ala)	1/11 (9%)			1/39 (3%)
c.1637A>G p.(Gly546Arg)		1/15 (7%)		1/39 (3%)
c.1636C>A p.(Gly546Lys)		1/15 (7%)		1/39 (3%)
c.1633G>A p.(Glu545Lys)		2/15 (13%)		2/39 (5%)
c.1624G>A p.(Glu542Lys)	1/11 (9%)	3/15 (13%)	2/13 (15%)	6/39 (15%)
c.1357G>A p.(Glu453Lys)			2/13 (15%)	2/39 (5%)
c.1252G>A p.(Glu418Lys)	1/11 (9%)			1/39 (3%)
c.1132T>C p.(Cys378Arg)			1/13 (8%)	1/39 (3%)
c.1049A>G p.(Asp350Gly)	1/11 (9%)			1/39 (3%)
c.1038_1040dup p.(Val346_Asn347insLys)	1/11 (9%)			1/39 (3%)
c.1035T>A p.(Asn345Lys)		1/15 (7%)		1/39 (3%)
c.328_330delGAA p.(Glu110del)	1/11 (9%)	1/15 (7%)	1/13 (8%)	3/39 (8%)
c.241G>A p.(Glu81Lys)	1/11 (9%)			1/39 (3%)
**Mean start BMI (adults ≥16 years) kg/m** ^**2**^	32.6 (24.9–41.8)	24.1 (20.2–25.6)	38.8 (27.0–49.4)	31.8 (20.2–49.4)
**Mean start BSA m** ^**2**^ **(children <16 years)**	1.09 (0.63–1.51)	1.13 (0.61–1.44)	0.97 (0.62–1.36)	1.06 (0.61–1.51)
**Site of overgrowth**				
Generalized	3/11 (27%)	2/15 (13%)	3/13 (23%)	8/39 (21%)
Facial	1/11 (9%)	0	3/13 (23%)	4/39 (10%)
Upper limb only	1/11 (9%)	0	0	1/39 (3%)
Lower limb only	3/11 (27%)	7/15 (47%)	4/13 (31%)	14/39 (36%)
Upper and lower limbs	0	1/15 (7%)	2/13 (15%)	3/39 (8%)
Thoracic region only	0	0	0	0
Thoracic region and limbs	2/11 (9%)	1/15 (7%)	0	3/39 (8%)
Truncal region only	0	0	0	
Truncal region and limbs/thoracic region	1/11 (9%)	3/15 (20%)	7/13 (54%)	11/39 (28%)
**Vascular/venous malformations**	2/11 (18%)	11/15 (73%)	10/13 (77%)	23/39 (59%)

*BMI* body mass index, *BSA* Body Surface Area

The main exclusion criteria were use of sirolimus in the prior 4 weeks; active skin infections; major surgery in the prior 12 weeks; pregnancy and lactation; live vaccines in the 4 weeks prior to, during, and 6 weeks after dosing; insufficient renal, hepatic, or bone marrow function; or prior/intercurrent HIV, hepatitis B/C, or tuberculosis infection. In the United Kingdom and France, participants with a blood neutrophil count of <1.0 × 10^9^/l were excluded; in the United States, participants with a count of <1.5 × 10^9^/l, or <1.0 × 10^9^/l and a diagnosis of benign ethnic neutropenia were excluded.

Participants were withdrawn if they or their parents requested, or if adverse events (AEs) of grade ≥3 occurred including neutropenia (<1.0 × 10^9^/l in France and the United Kingdom, <0.5 × 10^9^/l in the United States); proteinuria (e.g., 3+ on qualitative testing); liver dysfunction (alanine transaminase [ALT] >3 times the upper limit of normal); renal dysfunction (glomerular filtration rate [GFR] <70 mls/min/1.73 m^2^); pneumonitis or decline in respiratory reserve; severe, recurrent infections or septicemia; QTc prolongation; or other side effects at the discretion of the lead investigator at each site.

### Dosing regimen

Pharmacokinetic data for sirolimus for children and adults with renal transplants informed dosing algorithms.^19^ A target sirolimus plasma concentration of 2–6 ng/ml was selected based on in vitro preclinical studies^20^ off-label clinical experience,^13^ and with the aim of minimizing AEs. Given the known immunosuppressive effects of high-dose sirolimus, the lack of evidence that these doses are more efficacious in PROS, and the likely need for lifelong therapy we titrated dosing to achieve this target concentration. Steady state was defined as two consecutive concentrations within 10% of each other. Dosing and sirolimus monitoring regimens between the UK, France, and US sites are outlined in Supplemental Table S5. Treatment was paused during intercurrent infection, surgical intervention, or other AE when necessary.

### Biochemical evaluation

Laboratory assessments included blood cell counts, indices of renal function and liver function, fasting blood glucose and lipid profiles, and urinalysis including protein semiquantification. Sirolimus assays were performed in accredited clinical diagnostic laboratories.

### Quantification of overgrowth

Affected sites were defined by clinical observation. Unaffected sites showed no overgrowth, nor evidence of skin or vascular abnormalities. Unaffected sites compared with affected sites were (in order of preference) the contralateral limb/truncal region, a limb or trunk on the same side, or any other site without clear involvement. Those with generalized, but asymmetric overgrowth were deemed to have no unaffected site.

Dual energy X-ray absorptiometry (DXA) scans were performed using the same orientation and iDXA GE Lunar scanner for each participant at 0, 26, and 52-week time points. DXA imaging provides reproducible measurement of regional and whole body composition (bone, fat, and lean mass),^21^ though it cannot assess vascular or lymphatic malformations. We used DXA to evaluate the primary outcome measure in view of its wide applicability, its acceptability to children, and its capacity to generate concurrent estimates of unaffected and affected tissue sites. For adults too large to scan completely with one scan (*n* = 3), a second scan was performed to give full body coverage. Soft tissue volumes were obtained for total body and various body segments (left leg, right leg, right trunk, left trunk, right arm, left arm, and head) by converting masses to volumes assuming fat density of 0.9 g/ml and lean mass density of 1.1 g/ml.^22^ Total tissue volume included lean and fat volume, but not bone. Results were generated using the same, standardized iDXA GE Lunar software at each center.

T1-weighted magnetic resonance imaging (MRI) scans without contrast were also acquired in a subset of participants at 0, 26, and 52 weeks using the same scanner at each site (GE GEMR450 wide in the United Kingdom, IRM Siemens Magnetom Aera 1.5T in France, and 3T MRI scanner [Trio, Siemens Medical Solutions] in the United States). Scanning covered bony anatomical landmarks at proximal and distal ends of the target area, and an oblique scan plane of 5 mm thickness with up to 100 slices was used. All scans were blinded prior to analysis. For volume calculation, IDEAL fat (Dixon sequence) images were visualized using volumetric software (SliceOmatic, TomoVision, Magog, Canada). Morphology segmentation was performed through computation of watershed gradients. Tissues (fat, muscle, bone, and blood vessel) were manually defined and software was used to generate a surrogate of tissue volume using five slices, with manual adjustments where required.

### Evaluation of vascular lesions

Clinical photography was performed in participants with visible vascular lesions before and after sirolimus treatment using the same camera in the same room with consistent lighting and color balance. Unblinded assessment by a study investigator was undertaken using the following scoring system: –1 = worsening; 0 = no change; 1 = partial resolution; 2 = complete resolution. Vascular lesions were scored by hue, with annotation regarding the presence of lymphatic blebs.

### Quality of life assessment

Validated quality of life (QOL) questionnaires were administered before and after treatment (WHOQOL-BREF questionnaire for adults,^23^ and age-appropriate PedsQL questionnaires for children and parents^24,25^). The WHOQOL-BREF assesses four domains (physical, psychological, social, environmental).^26,27^ The PedsQL Generic Core Scales assess four domains (physical, emotional, social, and school) and two composite scores (physical, psychosocial), which were transformed to a 0–100 scale (25).

### Outcome measures

The primary outcome measure was percentage change in volume of measured affected and unaffected areas over treated and untreated periods. Fat, lean, and total (fat plus lean) tissue volumes were determined. Both DXA and volumetric MRI scanning were undertaken where possible and anatomically appropriate. Prior to analysis, DXA was selected for the primary outcome measure as DXA results were available in all participants whereas MRI was only performed in a subset. Secondary outcome measures were steady-state sirolimus plasma concentration, mean sirolimus doses to achieve the target plasma concentration, and additional measures of efficacy including hospitalizations, surgical interventions, and QOL scores before and after treatment.

### Statistical analyses

Intention-to-treat analyses were based on all enrolled participants. As-treated analyses were restricted to those who completed the study. Absolute volumes of affected and unaffected tissue at week 0 (designated X), week 26 (designated Y), and week 52 (designated Z), were compared. Tissue volume changes (week 0–26 and week 26–52) were designated “DELTA,” and the percent change “% Change.” Percent change for the untreated period was [100(Y–X/X)], and for the treated period [100(Z–Y/Y)]. Paired comparisons of mean volumes and mean changes in volumes during untreated ad treated periods were performed using single-sample, paired Student’s *t* test in SAS version 9.4, with confirmation of equal variances. A post hoc subanalysis was performed to examine the difference % change in tissue at affected and unaffected sites during the treatment phase in comparison with the run-in phase according to age, genotype, and phenotypic characteristics. Linear regression and analysis of variance (ANOVA) analyses were performed to evaluate statistical significance. Additional statistical analyses of normally distributed data of equal variance were performed using single-sample Student’s paired *t* tests and chi-squared analyses for discontinuous data.

### Safety analysis

Safety endpoints were adverse events (AEs) identified by laboratory testing, clinical examination, or self-report. Severity was graded with the Common Terminology Criteria for Adverse Events (CTCAE) (version 4.0). AEs were coded by the current version of the Medical Dictionary for Regulatory Activities (MedDRA), and the type, incidence, severity, and relationship to sirolimus were summarized by MedDRA System Organ Class and Preferred Term. Serious adverse events (SAEs) were defined as those that led to death, were life-threatening, necessitated hospital admission or prolongation of an admission, or that resulted in persistent or severe disability or incapacity, congenital anomalies, or any other medically important events. All AEs and SAEs possibly, probably, or definitely related to sirolimus were reviewed by an international committee composed of members of the study teams from the United Kingdom, France, and the United States.

## Results

### Study participants and disposition

**Fig. 2:**
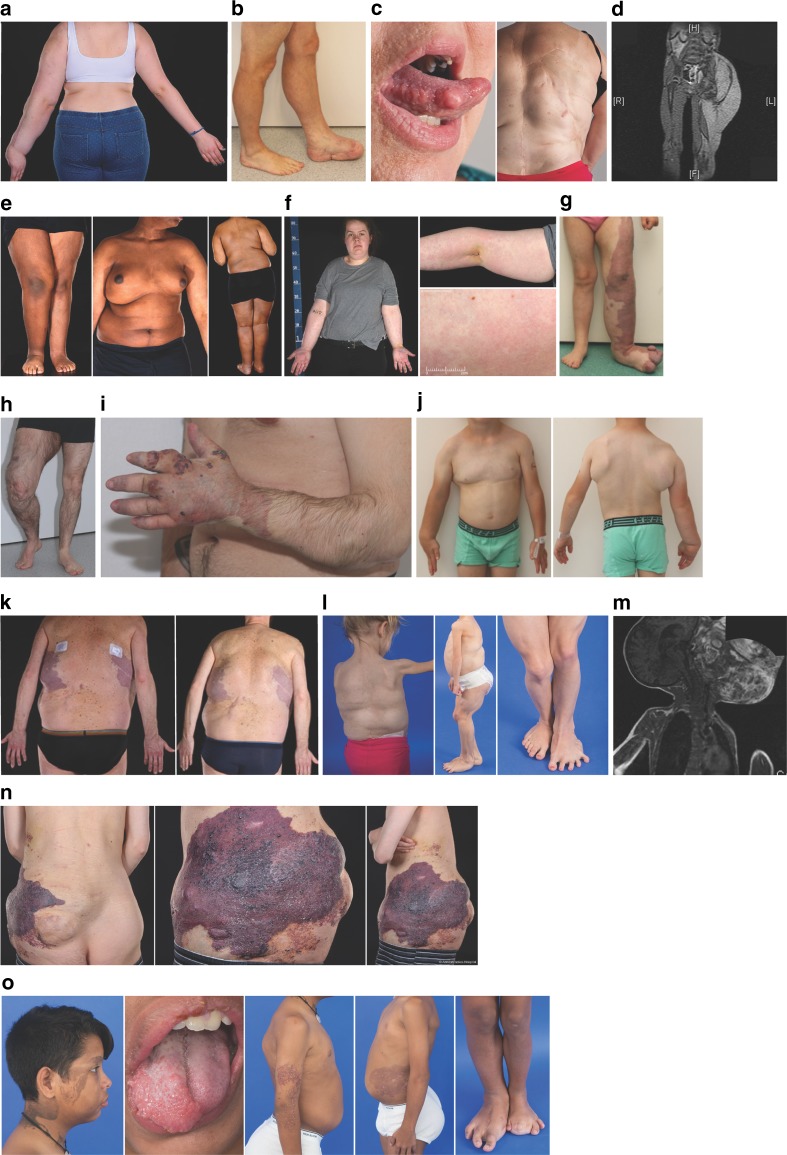
**Clinical heterogeneity of study participants with PIK3CA-related overgrowth spectrum.** **a** 15-year-old girl with fibroadipose hyperplasia of left arm; unaffected right arm (patient 1). **b** 39-year-old man with fibroadipose and bony hyperplasia of left lower leg, foot s/p multiple amputations, and lipectomies; unaffected right leg, foot (patient 17). **c** 48-year-old woman with tongue fibromas, fibroadipose hyperplasia masses of trunk, back, paraspinal s/p (status post) excision, and scoliosis s/p surgical bracing (patient 30). **d** 5-year-old boy with fibroadipose hyperplasia of the left leg, buttock visualized on magnetic resonance image (MRI) scan (patient 4). **e** 17-year-old boy with right fibroadipose hemihyperplasia (patient 2). **f** 19-year-old woman with right fibroadipose hemihyperplasia and capillary malformation, MCAP (Megalencephaly-capillary malformation syndrome) phenotype (patient 3). **g** 6-year-old girl with vascular malformations/overgrowth of left leg, foot; unaffected right leg, foot (patient 19). **h** 26-year-old man with vascular malformations/overgrowth of right leg; unaffected left leg (patient 12). **i** 23-year-old man with vascular malformations of the left arm and hand (patient 13). **j** 3-year-old boy with fibroadipose hyperplasia of the right arm, hand, fingers, and trunk and left partial chest/axilla (patient 16). **k** 44-year-old man with fibroadipose hyperplasia and vascular malformations of his back and bilateral legs (patient 10). **l** 8-year-old girl with fibroadipose hyperplasia masses of her trunk, back, paraspinal s/p excision, muscular hyperplasia of her legs and feet (left > right), regional lipohypoplasia of her bilateral arms (patient 27). **m** 6-year-old boy with facial infiltrating lipomatosis affecting left cheek visualized on MRI scan (patient 9). **n** 31-year-old man with vascular malformations and fibroadipose hyperplasia of the lower trunk, leg (patient 5). **o** 13-year-old boy with epidermal nevus of right face, neck; right facial, tongue, trunk, bilateral legs, feet (right > left) fibroadipose hyperplasia s/p multiple amputations/debulking surgeries of feet and toes; lymphovascular malformations of bilateral arms (right > left), trunk (patient 29). Written informed consent was obtained for use of all identifiable images.

Forty-one participants were screened and 39 enrolled, including 22 children (<16 years old) and 17 adults (≥16 years old) (Fig. 1b). The mean age was 16.6 years (SD 11.0, range 3–48); 44% were female and 56% male. Clinical characteristics of these participants are detailed in Supplementary Table S2, and representative images illustrating the overgrowth heterogeneity are shown in Fig. 2. Thirty participants completed 26 weeks of dosing, 2 discontinued the study after 23 weeks of dosing due to a temporary pause in dosing at one of the sites, and 7 were withdrawn due to AEs (Fig. 1b) (Supplementary Table S3). Of the 30 who completed 26 weeks dosing, 97% received >80% of their daily doses.

### Sirolimus plasma levels and dosing

The mean plasma sirolimus level recorded across the study in both adults and children was 3.4 ng/ml (95% confidence interval [CI] 3.1, 3.7), and the median level 3.3 ng/ml (25th/75th centiles 2.3, 4.2). The mean total daily sirolimus dose in adults was 1.2 mg once daily in capsules (95% CI 1.1, 1.3), and for children was 0.58 mg twice daily in oral solution (95% CI 0.50, 0.65).

### Efficacy-based outcome measures

**Fig. 3:**
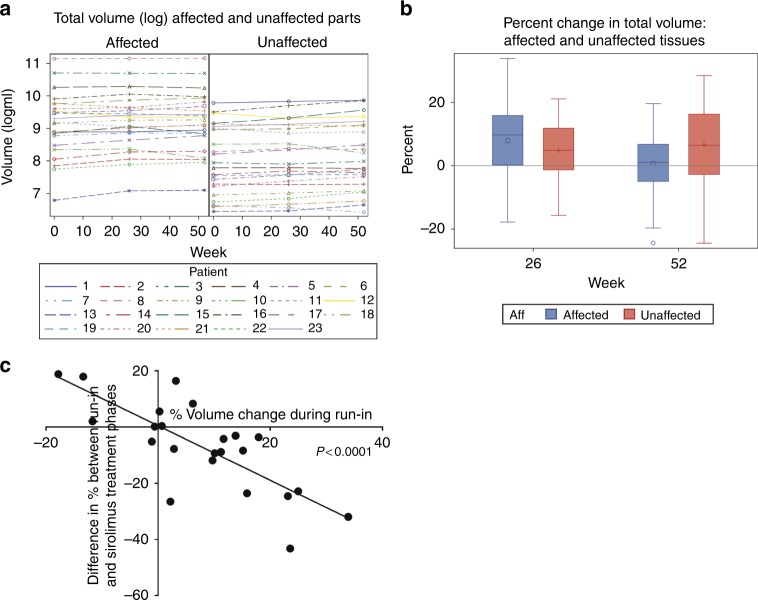
**Dual energy X-ray absorptiometry (DXA)-estimated changes in tissue volume during study period.** **a** Change in absolute volumes in ml at affected and unaffected sites at the beginning of the run-in period (week 0), after the 26-week run-in period (week 26), and after the 26-week sirolimus treatment period (week 52). Note major differences in the magnitude and variability of volume measurements between affected and unaffected sites. **b** Change in percent tissue volume at affected and unaffected sites during the run-in period at week 26 and during the sirolimus treatment phase at week 52. Individual values plotted, bars denote mean values and error bars standard deviation. **c** Post hoc subanalysis of progressive growth during the run-in period. Linear regression analysis of % volume change during run-in period, i.e., whether or not subjects had progressive overgrowth versus difference in % volume change between the run-in and treatment phase at affected sites. The result was significant implying the largest effect sizes were obtained in subjects with progressive overgrowth during the run-in period.

Volumes of fat and lean components of affected and (where possible) unaffected soft tissue body regions were measured using DXA at 0, 26, and 52 weeks (Fig. 3a). The anatomy of 23 participants permitted comparison of affected versus unaffected. Absolute tissue volumes were greater and more variable at affected versus unaffected sites as expected; at week 0 median total tissue volume at affected sites was 10,132 ml (interquartile range [IQR]: 4980, 17,055) and 3013 ml (IQR: 1465, 8599) at unaffected sites. In the untreated run-in period, the mean increase in total tissue volume at affected sites was +7.9% (SD 12.8) versus +4.8% (SD 9.7) at unaffected sites, (*p* = 0.19; SD 10.9) (Fig. 3b).

For the primary outcome measure, a significant reduction of –7.2% (SD 16.0; *p* = 0.04) was observed in the volume change of affected tissues during sirolimus treatment. In contrast, there was no significant increase for unaffected tissue (+1.7%, SD 11.5; *p* = 0.48). A significant reduction in lean tissue (muscle, connective tissue, and vasculature) of –6.3% (SD 13.3; *p* = 0.03) and a trend toward reduction of fat of –9.8% (SD 24.4; *p* = 0.07) was observed at affected but not unaffected sites during treatment (Supplementary Table S4). Viewing individual data points on scatter plots comparing volume changes in treated and untreated phases reinforces this conclusion (Supplementary Figure S1).

A post hoc subanalysis was performed to assess differences in response by age, clinical phenotype (qualitatively predominant adipose overgrowth, vascular overgrowth, or muscular/bony overgrowth), genotype, or overgrowth rate during the run-in period (Supplementary Figure S2). A significant correlation between the magnitude of treatment effect (difference between tissue volume changes in treatment and run-in periods) and of progressive overgrowth during the run-in period was seen (Fig. 3c). In addition, there was a trend toward greater tissue volume reduction in participants with predominant adipose overgrowth (–8.9% [SD 14.9], *n* = 17/23) than in participants without predominant adipose overgrowth (–2.4% [SD 19.4], *n* = 6/23, *p* = 0.14). There were no apparent differences with respect to age, genotype, or the presence/absence of vascular/venous malformations.

Serial MRI analyses of tissue volume were available for only 10/20 participants due to the site of overgrowth not being amenable to MRI scanning, or due to insufficient quality of acquired images for analysis. Technical challenges included variation in participant position and movement, varying slice thickness between scans, and anatomical factors such as fat investiture into muscles, preventing clear definition of tissue planes (Supplementary Figure S3**)**. Only six of ten MRI series that were eligible for analysis included both affected and unaffected sites. No statistically significant differences in tissue volume changes between the treatment phase and run-in phase were seen for either affected sites (–3.7% [SD 12.3]; *p* = 0.37) or unaffected sites (0% [SD 16.7]; *p* = 0.99).

### Secondary outcome measures

No differences were detected in QOL scores before and after sirolimus treatment (Supplementary Table S5) among adults or children. During run-in, five hospitalizations in five participants and two surgical interventions in two participants were recorded. In the treatment phase 15 hospitalizations in 9 participants and no surgical interventions arose. This difference was not significant (*p* = 0.24). Clinical photographs were evaluated for eight participants with superficial capillary or venous malformations and none changed during treatment. Five participants had lymphatic malformations and of these, two showed evidence of improvement with decreased superficial lymphangiectasis (Supplementary Figure S4), with three unchanged.

### Safety and tolerability

Twenty-eight of 39 participants (72%) had at least one AE deemed possibly, probably, or definitely related to sirolimus (Table 2). Twenty-one serious AEs (SAEs) (Supplementary Table S6) occurred among 12 participants during either run-in or treatment periods, and 7 had AEs leading to sirolimus discontinuation. The most common class of AE was infection (16/39 [41%] participants), followed by blood or lymphatic disorders (8/39 [21%]) (Supplementary Table S7). Clinically important AEs included grade 4 neutropenia (neutrophil count 0.02 × 10^9^/L), interstitial pneumonitis, and sirolimus hypersensitivity syndrome. This last SAE presented with prolonged fever and resulted in hospitalization for more than 6 weeks. Sirolimus was withdrawn in all three participants, all of whom subsequently made full recoveries. There were no significant changes in laboratory assessments including lipid profiles and fasting glucose concentrations in the treatment period.

**Table 2:** Overview of adverse events (AEs) and serious adverse events (SAEs)

	Intention-to-treat population (*n*=39)
Total number of AEs recorded	103 AEs in 31/39 (79%) participants 54/103 AEs (52%) unrelated to sirolimus
At least one sirolimus-related AE (all grades)	28/39 (72%)
At least one event of ≥ grade 3 severity	13/39 (33%)
Death (grade 5)	0 (0%)
At least one SAE	11/39 (28%)
At least one SAE and ≥ grade 3 severity	10/39 (26%)
At least one event leading to permanent discontinuation of sirolimus	7/39 (18%)

## Discussion

In this study 26 weeks of low-dose sirolimus therapy was associated with a small, but significant reduction in tissue growth at overgrown sites in participants with PROS. There was no measured effect on QOL or the overall extent of PROS-related skin lesions. These findings bolster the notion that sirolimus may be beneficial in PROS. One aim of this study was to inform selection of endpoints, and the powering and design of such trials.

Overgrowth in PROS comprises admixture of adipose tissue, vascular tissue, and bone. We anticipated that adipose and vascular tissues would be the most likely to respond to short-term treatment, given their natural capacity for rapid growth in the face of positive energy balance or injury. Furthermore, as these tissues can also regress physiologically, and as *PIK3CA* is involved in both cell proliferation and suppression of apoptosis, shrinkage of previously overgrown tissue rather than just deceleration of pathological overgrowth seemed a conceivable outcome of therapy. Using DXA body composition analysis we did not document evidence of significant and selective regression of affected tissue; however, overgrowth during run-in was associated with greater reduction in tissue volume during treatment. We also set out to use volumetric MRI to quantify adipose tissue volumes; however, technical and analytic challenges and inconsistency in serial imaging compromised this. MRI may nevertheless still be useful with more rigorous control of positioning and slice thickness.

This study had several limitations. Despite genetic homogeneity, phenotypic heterogeneity among participants was considerable. Post hoc subanalyses based on phenotype were inconclusive due to small sample size. Formally establishing whether our findings are generalizable to all PROS components, including the minor end of the phenotypic spectrum, will require availability of large PROS cohorts in future studies. The high frequency of study visits during treatment and the nonrandomized, open-label design may have led to overestimation of AEs. Furthermore, the time taken for sirolimus plasma concentrations to reach steady state and interruption of treatment due to intercurrent AEs in some patients will have reduced drug exposure and thus any treatment effect. The QOL assessment was limited by small sample sizes, and use of a generic assessment tool that omitted pain and functional impairment scoring. Additional tools exist, but none are specifically tailored to the PROS spectrum. Finally, participants who had generalized but asymmetric overgrowth were not included in the primary outcome analysis as an unaffected site could not be identified. In other patients, the “affected” and “unaffected” body regions we studied were defined based on clinical and radiographic appearances. However, the presence of variant-positive cells in apparently unaffected sites cannot be excluded, and thus growth at these sites could be at least partly pathologic.

Our findings suggest that, in the 26-week period assessed, sirolimus exerted its greatest effect on actively growing tissue, rather than inducing regression of prior overgrowth. Sirolimus did not significantly reduce tissue growth in unaffected areas, suggesting that sirolimus does not inhibit normal cellular growth at low doses. Subanalyses suggested a greater response in participants with predominantly adipose overgrowth; however, only the effect on lean tissue volume, assessed by DXA, reached significance. We cannot rule out greater effects of higher doses of sirolimus or longer durations of therapy; however, the effect size we observed will be of value in powering future studies.

A recent study by Adams et al.^17^ assessed a higher dose of sirolimus in a heterogeneous cohort with primarily vascular malformations. No genetic characterization was reported, but some were likely to have PROS based on clinical phenotypes. In that study, disease response (complete or partial) was defined with respect to radiologic imaging or QOL measures. No participant had complete response, and 87% had partial response, in some cases due only to changes in QOL measures. That study had limitations of likely heterogeneous etiology, no control or run-in period was used, affected and unaffected sites were not compared, and various imaging modalities were employed.

Medical therapy for PROS is likely to be suppressive rather than curative, and may be indefinite from childhood. Mindful of this, and of evidence that low concentrations of mTOR inhibitors are sufficient ex vivo to suppress basal hyperactivation of PI3K in cells from people with PROS,^12,20^ we used low-dose sirolimus to attempt to reduce the AE rate reported with higher doses. This yielded plasma concentrations below the immunosuppressive range used after organ transplantation. Nevertheless, a considerable number of AEs, several severe, were recorded, leading to 7 discontinuations of treatment, although 11/39 (28%) participants had no reported AEs. In comparison with Adams et al.^17^ we observed higher rates of discontinuation and infection-related AEs (41% in this study versus 15% [ref. ^17^] for CTCAE grade 2 or higher), but a lower incidence of hematological AEs (5% versus 27% [ref. ^17^] for CTCAE grade 3 or higher). In contrast, an AE profile that similar to ours was reported in a study of everolimus in tuberous sclerosis.^28^ There, 11/28 (39%) participants had a ≥ grade 3 event (versus 13/39 [33%] in our study) and the most frequent events were stomatitis 22/28 (79%), upper respiratory tract infection 22/28 (79%), and sinusitis 11/28 (39%). Discontinuation rates were only 1/28 (3.5%) in the first 6 months in the tuberous sclerosis trial. It is unclear whether some of the reported differences relate to the biology of PROS, to the heterogeneity of the participants in the prior studies, or whether they reflect a greater risk of infection with sirolimus therapy in PROS. Immunodeficiency is seen in several germline disorders featuring activated PI3K signaling, but in many patients with PROS the variant allele fraction in blood is undetectable or extremely low. We conclude that use of low-dose sirolimus in this study did not demonstrably decrease AE rates seen with higher-dose therapy.^29^

In summary, sirolimus has potential benefit for patients with PROS, especially where progressive adipose tissue overgrowth predominates. Randomized controlled trials with designs optimized for rare disease research are required to confirm this, and to establish the optimal therapeutic window, especially given our observed high rate of discontinuations despite the use of low-dose treatment. Based upon these results, sirolimus use in PROS should be evaluated on a case-by-case basis. Our study holds lessons for design of future trials, whether of mTOR inhibitors, or of trial agents targeting the *PIK3CA* gene product directly. The importance of objectively establishing the efficacy of sirolimus for PROS assumed new urgency while this study was in review, with the reporting of an uncontrolled, unregistered case series of patients with PROS treated with an experimental PIK3CA inhibitor on a compassionate basis.^30^ These anecdotal data suggested clinical efficacy without significant side effects; however, neither safety nor efficacy endpoints were prespecified. Testing of such agents in formal trials, perhaps against sirolimus, should be undertaken to permit objective, quantitative assessment of outcomes and AEs before unregulated use becomes widespread.

## Electronic supplementary material

Supplementary Material
