# Supplementary material for: Safety and efficacy of low-dose sirolimus in the PIK3CA-related overgrowth spectrum
Source: Genet Med. 2018 Oct 1;21(5):1189–98. doi: 10.1038/s41436-018-0297-9 (PMC6752269; doi:10.1038/s41436-018-0297-9)
Supplement: Supplementary file 1 — Supplementary Material [file 41436_2018_297_MOESM1_ESM.docx]

# **Supplementary Material**

**This PDF file includes:**

Supplementary Author List

Supplementary Tables S1 to S7

Supplementary Figures S1 to S4

## **PROMISE Working Group Authors**

**UK:** Boo Messahel^1^, Rachel Knox^2^, Jill Clayton-Smith^3^, Susan Huson^3^, Anna Martinez^4^, Sahar Mansour^5^, Kate Chandler^3^, Mary Glover^4^, Mehul Dattani^6^, Lynne Whitehead^7^, Mark Bolton^7^, Nick Fosh^8^, Martin Graves^9^

^1^Department of Paediatic Oncology, Cambridge University Hospitals NHS Trust

^2^Institute of Metabolic Science, Cambridge University Hospitals NHS Trust

^3^Division of Evolution & Genomic Sciences, Central Manchester Hospital NHS Trust

^4^Department of Dermatology, Great Ormond Street Hospital

^5^Department of Dermatology St George’s Hospital

^6^Department of Endocrinology, Great Ormond Street Hospital

^7^Pharmacy Department, Cambridge University Hospitals NHS Trust

^8^Wellcome Trust Clinical Research Facility, Cambridge University Hospitals NHS Trust

^9^Department of Medical Physics, Cambridge University Hospitals NHS Trust

**France:** Maud Carpentier^1^, Camille Fleck^1^, Amélie Cransac^2^, Agnes Maurer^3^, Carine Bonnin^3^, Pierre Potecher^4^, Alexandra Delignette^5^, Jean-Baptiste Rivière^6^, Valerie Corlier-Daire^7^

^1^Direction de la Recherche Clinique, University Hospital of Dijon, Dijon, France

^2^Department of Pharmacy, University Hospital of Dijon, Dijon, France

^3^Centre d'Investigation Clinique INSERM 1432, Centre Hospitalier Universitaire de Dijon, Dijon, Bourgogne, France

^4^Department of Interventional Radiology, Dijon University Hospital, Dijon, France

^5^Elithis Tower, Radiology, Dijon, France

^6^Equipe GAD UMR1231 et FHU TRANSLAD, CHU Dijon-Bourgogne et Université de Bourgogne, Dijon, France

^7^Département de Génétique, Institut Imagine, Université Paris-Descartes-Sorbonne-Paris Cité, hôpital Necker-Enfants malades, Paris, France

**US**: John M. Graham, Jr.^1^, Laura L. Tosi^2^, Courtney Duckworth^3^, Scott M. Paul^4^, Brooks P. Leitner^5^, Anjali Taneja^6^

^1^Cedars-Sinai Medical Center and Harbor-UCLA Medical Center, Los Angeles, CA

^2^Pediatric Orthopedics (LLT), ^3^Genetics and Metabolism Clinic (CD), Children's National Health System

^4^Rehabilitation Medicine Department, NIH Clinical Center

^5^Diabetes, Endocrinology, and Obesity Branch, Intramural Research Program, National Institute of Diabetes and Digestive and Kidney Diseases, National Institutes of Health, Bethesda, MD

^6^Clinical Genomics Section, Medical Genomics & Metabolic Genetics Branch, National Human Genome Research Institute, National Institutes of Health, Bethesda, MD

| **Design/Procedure** | **UK** | **US** | **France** | **Impact** |
| --- | --- | --- | --- | --- |
| **Run-in** | Inclusion criterion of DXA/MRI scan in preceding 26 weeks and enrolled just prior to sirolimus dosing | Enrolled at beginning of run-in period | Enrolled at beginning of run-in period | Hospitalisations/surgical data collected retrospectively  AE data during run-in collected retrospectively |
| **Start dose algorithm** | 0.5mg/m^2^ bd  (< 17y)  1mg od (>17 y) | 1mg/m^2^ bd (< 18 y)  <1.5m^2^ BSA 0.5 mg od  > 1.5 m^2^ BSA 1mg od | 0.5mg/m2 bd  (< 17y)  1mg od (>17 y) | Mean/median sirolimus levels and doses may be higher in US group |
| **Safety stopping criteria** | At site/sponsor discretion | Stop trial if grade 4 AE | At site/sponsor discretion |  |
| **Exclusion criterion – neutrophil count** | < 1.0 X10^^9^ | < 1.5 x10^^9^  < 1.0 x 10^^9^ if benign ethnic neutropenia | < 1.0 x10^^9^ |  |
| **QOL Qs** | PedsQL  WHO-QOL-Bref | PedsQL  WHO-QOL-Bref  Pain questionnaire | PedsQL  WHO-QOL-Bref  Pain questionnaire | No results on pain from UK site |
| **Imaging timing** | 0, 26, & 52 weeks | 0, 26, 38 & 52 weeks | 0, 26, & 52 weeks | Extra scan at 38 weeks in US; data not included in analysis |

**Table S1: Differences between UK, French and US site protocols**

| **Patient Designation** | **1** | **2** | **3** | **4** | **5*** | **6** | **7** | | **8** | **9** | **10** | **11** |
| --- | --- | --- | --- | --- | --- | --- | --- | --- | --- | --- | --- | --- |
| PIK3CA mutation | p.His1047Arg | p.Asp350Gly | p.Glu418Lys | p.His1047Arg | p.Glu542Lys | p.Val346_Asn347_Ins_Lys | p.Thr1025Ala | | p.His1047Arg | p.His1047Arg | p.Glu110_del | p.Glu81Lys |
| Original summary phenotype | FAH | MCAP | MCAP | FAH | FAH | FAH | CLOVES | | FAH | Infiltrating facial lipomatosis | CLOVES | MCAP/  FAH |
| Age at time of evaluation | 14 | 17 | 19 | 5 | 31 | 37 | 13 | | 12 | 6 | 44 | 19 |
| Age at onset of symptoms | 0 | 0 | 0 | 0 | 0 | 0 | 0 | | 0 | 0 | 0 | 0 |
| Sex | F | M | F | M | M | M | F | | M | M | M | F |
| Epidermal Nevus | N | Y | N | N | N | Y | N | | N | N | Y | N |
| Fibro adipose Overgrowth | Y | Y | Y | Y | Y | Y | Y | | Y | Y | Y | Y |
| Affected areas of overgrowth | Left arm and shoulder | R HH and head | R HH and head | Left leg and buttock | Trunk and right leg | Left leg | Right leg and both feet | | Right leg and foot | Left cheek | Back and legs | Right HH, right foot and toe |
| Regional lipo hypoplasia (affected areas) | No | No | No | No | No | Yes | No | | No | No | No | No |
| Vascular Malformations (one or more) | No | No | No | No | Yes | Yes  (right leg) | Yes | | No | No | Yes | Yes |
| Polydactyly | No | No | No | No | No | No | No | | No | No | No | No |
| Syndactyly | No | No | No | No | No | Yes | No | | Yes | No | No | No |
| Kidney abnormalities | No | No | No | No | No | No | No | | No | No | No | No |
| Other malformations | No | No | No | No | No | No | No | | No | No | No | No |
| Other skin abnormalities |  |  |  |  |  |  |  | |  |  |  |  |
| **Natural History Features** | | | | | | | | | | | | |
| General Growth BSA m^2^ or BMI kg/m^2^ | BMI 32.4 | BMI 37 | BMI 48.1 | BSA 0.63 | BMI 25.7 | BMI 27.8 | BSA 1.46 | BSA 1.52 | | BSA 0.78 | BMI 33.1 | BMI 24.1 |
| OFC (centile) | N/A | >95^th^ | >95th | N/A | N/A | N/A | N/A | N/A | | N/A | N/A | >95^th^ C |
| Development | Normal | Global delay | Mild learning difficulty | Normal | Normal | Normal | Normal | Normal | | Normal | Normal | Normal |
| Surgeries (debulking or amputation) | >3 debulking surgeries | None | None | 1 debulking surgery | >2 debulking surgeries | Multiple debulking/ liposuction | None | >7 debulking surgeries | | 1 debulking surgery | >2 debulking surgeries | N/A |
| **Response to treatment % change in volume during 26-week run-in period (week 26) and during 26 week sirolimus treatment period (week 52)** | | | | | | | | | | | | |
| Affected (week 26) | 0.3 | -2.4 | -1.3 | 23.1 | -0.6 | 2.8 | 6.2 | 11.7 | | 9.7 | 0.7 | N/A |
| Affected (week 52) | 5.8 | -7.2 | -15.9 | -1.4 | -0.5 | -4.9 | 14.5 | 7.5 | | -2.2 | 1.0 | N/A |
| Unaffected (week 26) | 4.9 | N/A | N/A | -1.3 | -3.9 | 0.7 | 14.5 | 2.5 | | -6.2 | 11.2 | N/A |
| Unaffected (week 52) | 3.4 | N/A | N/A | 1.3 | 8.6 | -2.8 | 20.8 | 13 | | -13.0 | -3.2 | N/A |

| **Patient Designation** | **12** | **13** | **14** | **15** | **16** | **17** | **18** | | **19** | **20** | **21** | **22** |
| --- | --- | --- | --- | --- | --- | --- | --- | --- | --- | --- | --- | --- |
| Mutation | p.Glu542Lys | p.Glu545Lys | p.Glu545Lys | p.Gln546Arg | p.His1047Arg | p.His1047Arg | p.His1047Leu | | p.Gln546Lys | p.Met1043Val | p.His1047Leu | p.Glu110_del |
| Original Summary Phenotype | KTS | KTS | KTS | KTS | FAH | Segmental overgrowth | KTS | | KTS | FAH | FAH | KTS |
| Age at time of evaluation | 26 | 23 | 19 | 16 | 3 | 39 | 11 | | 6 | 9 | 13 | 6 |
| Age at onset of symptoms | 0 | 0 | 0 | 0 | 0 | 0 | 0 | | 0 | 0 | 0 | 0 |
| Sex | M | M | F | F | M | M | F | | F | F | M | M |
| Epidermal Nevus | No | No | No | No | No | No | No | | No | Yes | No | No |
| Fibroadipose Overgrowth | No | No | No | No | Yes | No | No | | No | Yes | Yes | No |
| Affected Areas of Overgrowth | Right lower limb | Left upper limb | Left upper limb | Right lower limb | Right upper limb and fingers | Left lower limb and foot | Left lower limb and right foot | | Left lower limb and foot | Hands and toes | Thorax | Left lower limb and foot |
| Regional lipohypoplasia (Affected Areas) | No | No | No | No | No | No | Yes | | No | No | No | No |
| Vascular Malformations (one or more) | Yes | Yes | Yes | Yes | No | No | Yes | | Yes | Yes | No | Yes |
| Polydactyly | No | No | No | No | No | No | Yes | | No | No | No | No |
| Syndactyly | No | No | No | No | No | No | No | | No | No | No | Yes |
| Kidney abnormalities | No | No | No | No | No | No | No | | No | No | No | No |
| Other malformations | No | No | No | No | No | No | No | | Strabismus and scoliosis | No | No | No |
| Other skin abnormalities |  |  |  |  |  |  |  | |  |  |  |  |
| **Natural History Features** | | | | | | | | | | | | |
| General Growth BSA m^2^ or BMI kg/m^2^ | BMI 22.7 | BMI 27 | BMI 20.2 | BMI 25.2 | BSA 0.61 | BMI 25.6 | BSA 1.21 | BSA 0.69 | | BSA 1.41 | BSA 1.44 | BSA 0.92 |
| OFC (centile) | N/A | N/A | N/A | N/A | N/A | N/A | N/A | 51cm (M) | | N/A | N/A | N/A |
| Development | Normal | Mild learning difficulty | Normal | Normal | Normal | Normal | Normal | Psychomotor retardation | | Normal | Normal | Normal |
| Surgeries (debulk/amputation) | None | None | 1 amputation | None | 2 amputations 1 lipectomy | 4 amputations 2 lipectomies | 3 amputations  1 liposuction | None | | None | 2 lipectomies | 1 amputation |
| **Response to treatment % change in volume during 26-week run-in period (week 26) and during 26 week sirolimus treatment period (week 52)** | | | | | | | | | | | | |
| Affected (week 26) | -11.7 | 2.2 | -13.4 | -17.8 | 33.9 | N/A | N/A | 25.0 | | 23.6 | 15.6 | 17.9 |
| Affected (week 52) | N/A | N/A | N/A | 1.1 | 1.9 | N/A | N/A | 2.1 | | -19.7 | -7.7 | 14.3 |
| Unaffected (week 26) | -2.6 | 1.1 | -14.4 | -15.7 | 1.7 | N/A | N/A | 11.9 | | 17.9 | 21.1 | 14.5 |
| Unaffected (week 52) | N/A | N/A | N/A | 6.0 | 20.2 | N/A | N/A | -4.7 | | 28.4 | 17.0 | 16.3 |

| **Patient Designation** | **23** | **24** | **25** | **26** | **27** | **28** | **29** | **30** | **31** | **32** | **33** |
| --- | --- | --- | --- | --- | --- | --- | --- | --- | --- | --- | --- |
| PIK3CA Mutation | p.Asn1044Lys | p.His1047Arg | p.Asn345Lys | p.Glu542Lys | p.His1047Arg | p.His1047Arg | p.Glu110_ del | p.Glu542Lys | p.His1047Leu | p.His1047Arg | p.His1047Tyr |
| Original Summary Phenotype | KTS | Segmental overgrowth | FAH | FAH | CLOVES | FAH | CLOVES | CLOVES | CLOVES | FAH | CLOVES |
| Age at time of evaluation | 13 | 31 | 22 | 9 | 8 y | 10 y | 13 y | 48 y | 10 y | 5 y | 16 y |
| Age at onset of symptoms | 0 | 0 | 0 | 0 | Prenatal | 0 | 0 | 0 | 0 | 0 | 0 |
| Sex | M | M | F | F | F | M | M | F | F | F | M |
| Epidermal Nevus | No | No | No | No | Y | N | Y | Y | Y | N | Y |
| Fibroadipose Overgrowth | No | No | Yes | Yes | Y | Y | Y | Y | Y | Y | Y |
| Affected Areas of Overgrowth | Left lower limb and foot | Lower limbs | Thorax | Left lower limb | Bilateral legs, feet (L>R); Trunk | L leg, buttock, foot | Both legs, feet (R>L); L trunk, R face | R upper leg; L lower leg; R arm, hand; trunk, back; R face | Both legs (R>L), L buttock; R foot; abdomen | L trunk (chest); L leg; R foot, great toe | Both legs (L>R) & arms (R>L); L face |
| Regional lipohypoplasia (Affected Areas) | No | No | Yes | Yes | Y (Face, upper body) | N | N | N | Y (Upper body) | Y (Upper body) | N |
| Vascular Malformations (≥1) | No | No | No | No | Y | N | Y | N | N | N | Y |
| Polydactyly | Yes | No | No | No | N | Y (PPD foot) | N | N | N | N | N |
| Syndactyly | No | No | No | No | N | N | Y | N | N | N | N |
| Kidney abnormalities | No | No | No | No | N | Y; L pelvicaliesctasis | N | Y; cysts | Y; Bilateral nephro-blastomatosis | N | N |
| Other malformations | Medulla lipoma | No | Scoliosis | Tethered cord scoliosis | Cerebral infarcts | N | SNHL; scoliosis; R hydrocele, cystic mass | L Ovarian  Cyst; DVT R leg; scoliosis; splenic cysts, ↑R kidney; SNHL | N | N | HC |
| Other skin abnormalities |  |  |  |  |  | Hyper-/hypo-  Pigmentation; ↑hair & cutis marmorata L leg |  |  | Hyper-  pigmentation | N | Port-wine stain |
| Other skin abnormalities | **Natural History Features** |  |  |  |  |  |  |  |  |  |  |
| **Natural History Features** | | | | | | | | | | | |
| BMI (kg/m^2^)/ BSA (m^2^) | BSA 1.65 | BMI 29.7 | BMI 21.6 | BSA 1.10 |  | 15.4/0.98 | 22.7/1.01 | 42.9 | 23.5/1.3 | 16.2/0.77 | 35.8 |
| OFC (centile) | N/A | N/A | 54 cm (-1DS) | 51.5cm M- | <5th | 50th | 60th | 75-90th | 95th | 50th | >98th |
| Development | Normal | Normal | Normal | Normal | Speech delays | Normal, ADHD | Normal | Normal | Normal, ADHD | Normal | Delayed |
| Surgeries (debulk/amputation) | 1 lipectomy | 3 amputations | 2 liposuctions | 2 liposuctions | Y (4/0) | Y (3/5) | Y (2/4) | Y (6/0) | Y (5/1) | Y (0/2) | N |
| **Response to treatment % change in volume during 26-week run-in period (week 26) and during 26 week sirolimus treatment period (week 52)** | | | | | | | | | | | |
| Affected (week 26) | N/A | N/A | N/A | N/A | 10.1 | N/A | -1.1 | N/A | 3.2 | 13.8 | N/A |
| Affected (week 52) | N/A | N/A | N/A | N/A | 0.8 | N/A | -6.3 | N/A | 19.6 | 10.7 | N/A |
| Unaffected (week 26) | N/A | N/A | N/A | N/A | 6.3 | N/A | 3.8 | N/A | 17.8 | 7.5 | N/A |
| Unaffected (week 52) | N/A | N/A | N/A | N/A | 6.4 | N/A | 0.2 | N/A | 14.6 | 10.5 | N/A |

| **Patient Designation** | **34** | **35** | **36** | **37** | **38** | **39** |
| --- | --- | --- | --- | --- | --- | --- |
| PIK3CA Mutation | p.Cys378Arg | p.His1047Arg | p.Asn1044Lys | p.Glu453Lys | p.Glu453Lys | p.Glu542Lys |
| Original Summary Phenotype | CLOVES | FAH | CLOVES | FAH | MCAP | FAH |
| Age at time of evaluation | 3 y | 8 y | 20 y | 24 y | 11 y | 3 y 6 m |
| Age at onset of symptoms |  | 18 m | 12 m | 0 | 0 | 0 |
| Sex | M | F | M | F | M | M |
| Epidermal Nevus | N | N | Y | Y | N | N |
| Fibroadipose Overgrowth | Y | Y | Y | Y | Y | Y |
| Affected Areas of Overgrowth | L leg, foot; R arm, hand; R back | R leg, foot, 1,2 toes | R leg; Bilateral feet (R>L), R chest, abdomen, back | Bilateral legs, feet, buttocks | Bilateral legs, feet, arms, hands (L>R), L hemimegalencephaly | R leg, foot, trunk |
| Regional lipohypoplasia (Affected Areas) | Y (mild chest) | N | N | N | N | N |
| Vascular Malformations (one or more) | Y | N | Y | Y | Y | N |
| Polydactyly | N | N | N | N | N | N |
| Syndactyly | Y (toes) | N | N | N | Y | N |
| Kidney abnormalities | N | Y; hydronephrosis | N | N | Y | N |
| Other malformations | N | N | N | N | L hemimegalencephaly, Spinal vascular malformation | N |
| Other skin abnormalities | Hyperpigmented lesions; cutis marmorata | Macule, Capillary malformation | N | Hyperpigmented lesions | N | N |
| Other skin abnormalities |  |  |  |  |  |  |
| **Natural History Features** | | | | | | |
| BMI (kg/m^2^)/ BSA (m^2^) | 16.5/0.65 | 28.8/1.36 | BMI 27 | BMI 49.4 | BSA 1.3 | BSA 0.62 |
| OFC (centile) | 25-50^th^ | >98th | >98th | >95th | >95th | >95th |
| Development | Delays | Normal | LD, ADHD | Normal | Delays/ID | Normal |
| Surgeries (debulk/amputation) | None | None | 1/1 | 1/0 | 0/0 | 0/2 |
| **Response to treatment % change in volume during 26-week run-in period (week 26) and during 26 week sirolimus treatment period (week 52)** | | | | | | |
| Affected (week 26) | 15.1 | 11.2 | N/A | N/A | N/A | N/A |
| Affected (week 52) | 6.8 | 2.4 | N/A | N/A | N/A | N/A |
| Unaffected (week 26) | 10.3 | 7.5 | N/A | N/A | N/A | N/A |
| Unaffected (week 52) | 22.9 | 9.2 | N/A | N/A | N/A | N/A |

**Table S2:** **Details of genotype and phenotypic characteristics of enrolled participants(n=39).** Abbreviations: ADHD, attention deficit hyperactivity disorder, CLOVES = Congenital Lipomatous Overgrowth, Vascular Malformations, and Epidermal Nevi, DVT = deep vein thrombosis, FAH =fibroadipose hyperplasia, ID = Intellectual disability, KTS = Klippel-Trénaunay syndrome, LD = learning disability, MCAP = megalencephaly and capillary malformation syndrome, OFC = orbital frontal circumference, SNHL = Sensorineural hearing loss. Subjects #1-11 were recruited in the UK, subjects #12-26 in France, and subjects #27-39 in the US. *Subject 5 has a *GNA11* mutation (c.627G>T, p.Gln209His) in addition to a *PIK3CA* mutation

| **Patient Designation** | **11** | **12** | **13** | **14** | **18** | **27** | **34** |
| --- | --- | --- | --- | --- | --- | --- | --- |
| **PIK3CA mutation** | p.Glu81Lys | p.Glu542Lys | p.Glu545Lys | p.Glu545Lys | p.His1047Leu | p.His1047Arg | p.Cys378Arg |
| **Original summary phenotype** | MCAP/  FAH | KTS | KTS | KTS | KTS | CLOVES | CLOVES |
| **Age at time of evaluation** | 19 | 26 | 23 | 19 | 11 | 8 | 3.5 |
| **Sex** | F | M | M | F | 0 | F | M |
| **Affected areas of overgrowth** | Right HH, right foot and toe | No | No | No | F | Trunk (Abd, Back), Bilateral legs, feet | Right back, Right arm, hand, Left leg, foot |
| **Other skin abnormalities** |  | Right lower limb | Left upper limb | Left upper limb | No |  |  |
| **Surgeries (debulking or amputation)** | N/A | None | None | 1 amputation | No | 4 surgeries | N/A |
| **AEs** | Infections (2 x grade 3 AEs)  Headache | Right thigh superficial thrombosis + anemia + urinary infection | Hypertriglyceridemia | Superficial venous bleeding + abdominal pain + superficial phlebitis, node, left foot pain + sinusitis + pelvic thrombosis | Left lower limb and right foot | RLL infiltrate X3 (possible pneumonia) (grade 3); Interstitial pneumonitis (grade 3) | Neutropenia (grade 2); (grade 4 X1) |
| **SAEs** | Two hospitalisations: PID and cellulitis of the foot | Right cellulitis+Still's disease later diagnosed as sirolimus hypersensitivity syndrome | Pulmonary embolism-Chest pain | Anemia + acute appendicitis + hemarthrosis | 2 hospitalizations:  Enterovirus meningitis  Thigh stump cellulitis | 4 hospitalizations: pneumonia X3, pneumonitis X1 | Neutropenia (grade 4) |
| **Onset SAEs** | 4 weeks into treatment | 10 weeks into treatment | 8 weeks into treatment | Before sirolimus treatment | 4 and 8 weeks into treatment | 12, 16, 20, & 22 weeks into treatment | 18 weeks into treatment |
| **Withdrawn** | 6 weeks after commencing sirolimus | 10 weeks after commencing sirolimus | 8 weeks after commencing sirolimus | 16 weeks after commencing sirolimus | 8 weeks after commencing sirolimus | 22 weeks after commencing therapy | 18 weeks after commencing therapy |
| **Reason for withdrawal** | Recurrent infection | Sirolimus hypersensitivity syndrome | Pulmonary embolism | Anemia + acute appendicitis + hemarthrosis | Thigh stump cellulitis/ 2^nd^ hospitalisation | Interstitial pneumonitis | Neutropenia |

**Table S3:** **Characteristics of participants withdrawn due to sirolimus related adverse events**

|  | **Tissue Type** | **Mean percentage change in tissue volume (SD) N=23** | | | **P value for mean percent change** |
| --- | --- | --- | --- | --- | --- |
|  |  | **Run-in** | **Sirolimus** | **Δ** |  |
| **Affected** | **Total** | 7.9  (12.8) | 0.71  (10.2) | -7.2  (16.0) | 0.04 |
|  | **Fat** | 11.3  (17.5) | 1.5  (15.6) | -9.8  (24.4) | 0.07 |
|  | **Lean** | 6.3  (11.6) | -0.0  (8.9) | -6.3  (13.3) | 0.03 |
| **Unaffected** | **Total** | 4.8  (9.7) | 6.5  (12.3) | 1.7  (11.5) | 0.48 |
|  | **Fat** | 5.6  (12.0) | 8.0  (21.3) | 2.4  (19.6) | 0.57 |
|  | **Lean** | 4.8  (9.6) | 5.9  (13.0) | 1.1  (12.9) | 0.70 |

**Table S4. DXA-measured differences in tissue volume in affected and unaffected tissues.** Total tissue = lean + fatty tissue.

|  | **Physical Health** | **Psychological Health** | **Social Relationships** | **Environment** | **Physical and Psychosocial Health Total Mean Scores** |
| --- | --- | --- | --- | --- | --- |
| **Adults**  **n=9** | 0.25  (-8.9, 9.4)  p =0.95 | -1.25  (-7.3, 4.8)  p=0.64 | 6.3  (-1.8, 14.3)  p=0.11 | -7.3  (-32.5, 17.7)  p=0.51 | - |
| **Children**  **n=15** | - | - | - | - | -1.7  (-5.0, 8.8)  p=0.59 |
| **Parents**  **n=19** | - | - | - | - | 4.9  (-0.7, 10.4)  p=0.08 |

**Table S5: Quality of Life (QoL) scores during study** Results are shown as the change in QoL score (95% confidence boundaries; p-value)

|  | **Serious Adverse Events** | | | | | |
| --- | --- | --- | --- | --- | --- | --- |
| **Class** | | Description | CTCAE severity grade | Relatedness | Outcome | Led to Discontinuation of Sirolimus? |
| **Blood and lymphatic disorders** | | Anemia | 3 | Not related | Hospitalization | No |
|  |  | Anemia | 2 | Not related | Hospitalization | No |
|  |  | Neutropenic fever | 3 | Not related | Clinically important | No |
|  | | Neutropenia | 4 | Possible | Clinically important | Yes |
| **Gastrointestinal disorders** | | Constipation aggravated | 3 | Not related | Hospitalization | No |
| **Infections and infestations** | | Appendicitis | 3 | Not related | Hospitalization | No |
|  |  | Cellulitis of face | 3 | Possible | Hospitalization | No |
|  |  | Cellulitis of foot | 3 | Possible | Hospitalization | Yes |
|  |  | Epstein Barr Virus infection | 3 | Possible | Hospitalization | No |
|  |  | Cellulitis | 3 | Possible | Hospitalization | No |
|  |  | Cellulitis | 3 | Possible | Hospitalization | No |
|  |  | Cellulitis | 3 | Possible | Hospitalization | No |
|  |  | Cellulitis | 3 | Not related | Hospitalization | No |
|  |  | Pelvic infection | 3 | Possible | Hospitalization | No |
|  |  | Pneumonia | 2 | Not related | Hospitalization | No |
|  |  | Viral meningitis | 3 | Possible | Hospitalization | No |
| **Injury, poisoning and procedural complications** | | Over-medication | 1 | Definite | Clinically important | No |
| **Musculoskeletal and connective tissue disorders** | | Sirolimus hypersensitivity syndrome | 3 | Definite | Hospitalization | Yes |
| **Respiratory, thoracic and mediastinal disorders** | | Interstitial pneumonitis | 3 | Definite | Hospitalization | Yes |
| **Vascular disorders** | | Hemarthrosis | 2 | Possible | Hospitalization | No |
|  |  | Pulmonary embolus | 3 | Probable | Hospitalization | Yes |

**Table S6: Serious adverse events (SAEs) recorded during the study**

| **Class of AE** | **Grade** | **Possible** | **Probable** | **Definite** | **Total** |
| --- | --- | --- | --- | --- | --- |
| **Blood and lymphatic disorders** | 1 | 2/39 (5%) | 0 | 0 |  |
|  | 2 | 1/39 (3%) | 0 | 0 |  |
|  | 3 | 1/39 (3%) | 0 | 0 |  |
|  | 4 | 1/39 (3%) | 0 | 0 |  |
|  |  |  |  |  | **8/39 (21%)** |
| **Gastrointestinal disorders** | 1 | 2/39 (5%) | 0 | 0 |  |
|  |  |  |  |  | **2/35 (6%)** |
| **Infections and infestations** | 1 | 7/39 (18%) | 1/39 (3%) | 0 |  |
|  | 2 | 8/39 (21%) | 0 | 0 |  |
|  | 3 | 5/39 (13%) | 0 | 0 |  |
|  |  |  |  |  | **16/39 (41%)** |
| **Injury, poisoning and procedural complications** | 1 | 0 | 0 | 1/39 (3%) |  |
|  |  |  |  |  | **1/39 (3%)** |
| **Metabolism and nutrition disorders** | 1 | 0 | 0 | 3/39 (8%) |  |
|  |  |  |  |  | **3/38 (8%)** |
| **Musculoskeletal and connective tissue disorders** | 3 | 1/39 (3%) | 0 | 0 |  |
|  |  |  |  |  | **1/39 (3%)** |
| **Nervous system disorders** | 1 | 1/39 (3%) | 0 | 1/39 (3%) |  |
|  |  |  |  |  | **2/39 (5%)** |
| **Renal and urinary disorders** | 1 | 1/39 (3%) | 0 | 0 |  |
|  |  |  |  |  | **1/39 (3%)** |
| **Respiratory, thoracic and mediastinal disorders** | 1 | 1/39 (3%) | 0 | 0 |  |
|  | 3 | 0 | 0 | 1/39 (3%) |  |
|  |  |  |  |  | **2/39 (5%)** |
| **Skin and subcutaneous disorders** | 1 | 2/39 (5%) | 0 | 0 |  |
|  |  |  |  |  | **2/39 (5%)** |
| **Vascular disorders** | 1 | 1/39 (3%) |  |  |  |
|  | 2 | 1/39 (3%) |  |  |  |
|  | 3 |  | 1/39 (3%) |  |  |
|  |  |  |  |  | **3/39 (8%)** |

**Table S7.** **Breakdown of adverse events by system and grade.** Adverse events (AEs, grades 1-5) experienced at least once and deemed to have possible, probable, or definite relatedness to sirolimus. No clinically significant abnormalities in safety blood tests aside from those reported as AEs in individual participants (anemia, neutropenia, and dyslipidemia) and no clinically significant proteinuria were seen.


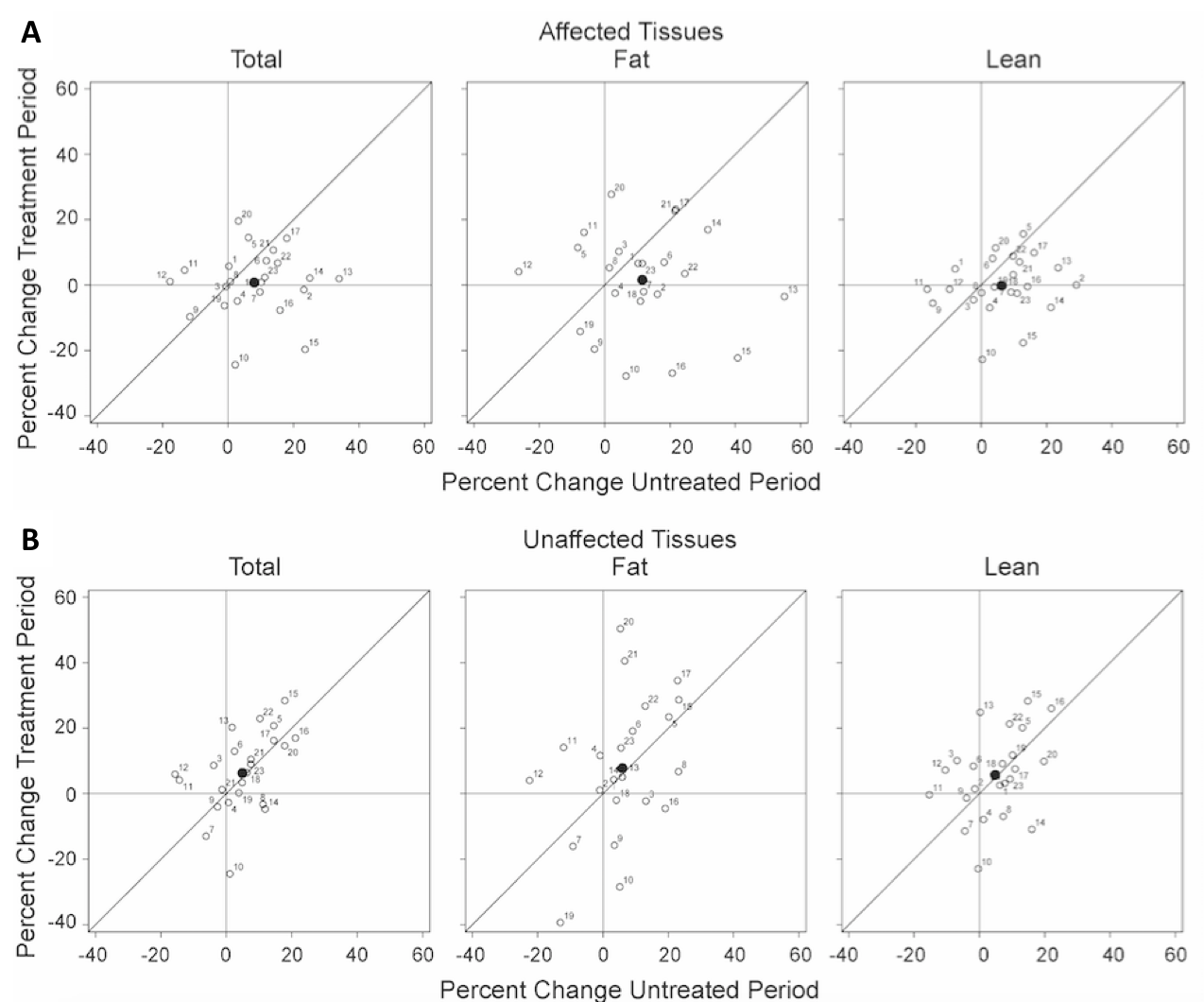


**Figure S1*:* Correlations between percent changes in tissue volumes During Treatment and Run-in Periods. A.** Scatter plots of percent changes in volumes for affected tissues in treated versus untreated periods. **B.** Scatter plot of percent change in tissue volume for unaffected tissues in treated versus untreated periods. Diagonal lines are lines of identity for reference purposes. Black dotes represent centroids. Points below the diagonal line illustrate those for which percent change in the treated period was less than the percent change in the untreated period (i.e., participants on sirolimus grew less than during the run-in period and there appeared to be a response to treatment).


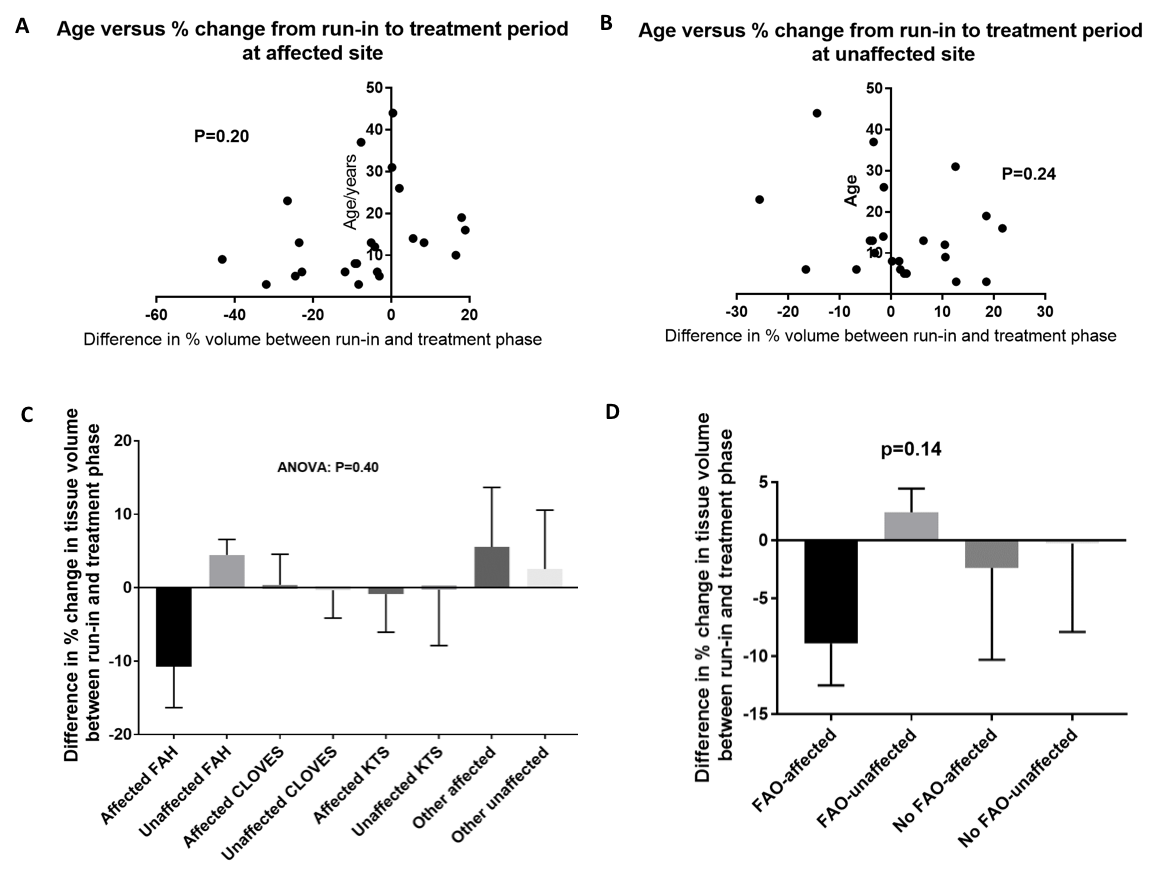


**
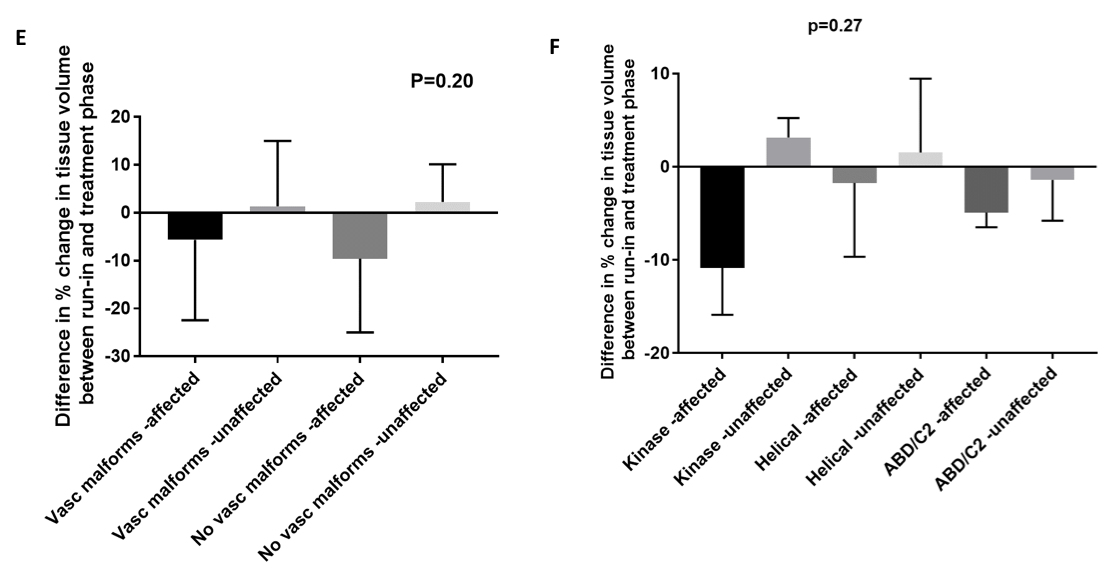
**

**Figure S2 *Post-hoc* sub-analyses of change in tissue volume according to selected demographic, phenotypic and genotypic features. A.** and **B**. Linear regression analysis of age and % change in tissue volume between run-in and treatment phase at **A.** affected and **B.** unaffected sites; No significant correlations were seen. **C.** Difference in percent change in tissue volume between run-in and treatment phase at affected and unaffected sites according to phenotype; FAH = Fibroadipose Hyperplasia (n=8); CLOVES = Congenital Lipomatous Overgrowth, Vascular Malformations, and Epidermal Nevi (n=6); KTS = Klippel Trenauney Syndrome (n=6); Other, n=3. Analysis was by ANOVA. Error bars = SEM. **D.** Difference in percentage change in tissue volume between run-in and treatment phase at affected and unaffected sites according to presence (n=17) or absence (n=6) of fibroadipose overgrowth (FAO). There was no significant difference in percent tissue change by ANOVA. Error bars = SEM. **E:** Difference in percent change in tissue volume between run-in and treatment phase at affected and unaffected sites according to presence (n=14) or absence (n=9) of vascular malformations. There was no significant difference by ANOVA. Vasc malforms = vascular malformations. Error bars = SEM. **F.** Difference in percentage change in tissue volume between run-in and treatment phase at affected and unaffected sites according to domain site of mutation within *PIK3CA*; kinase (n=12), helical (n=6), or ABD/C2 domains (n=5). No significant difference was seen by ANOVA. Error bars = SEM.


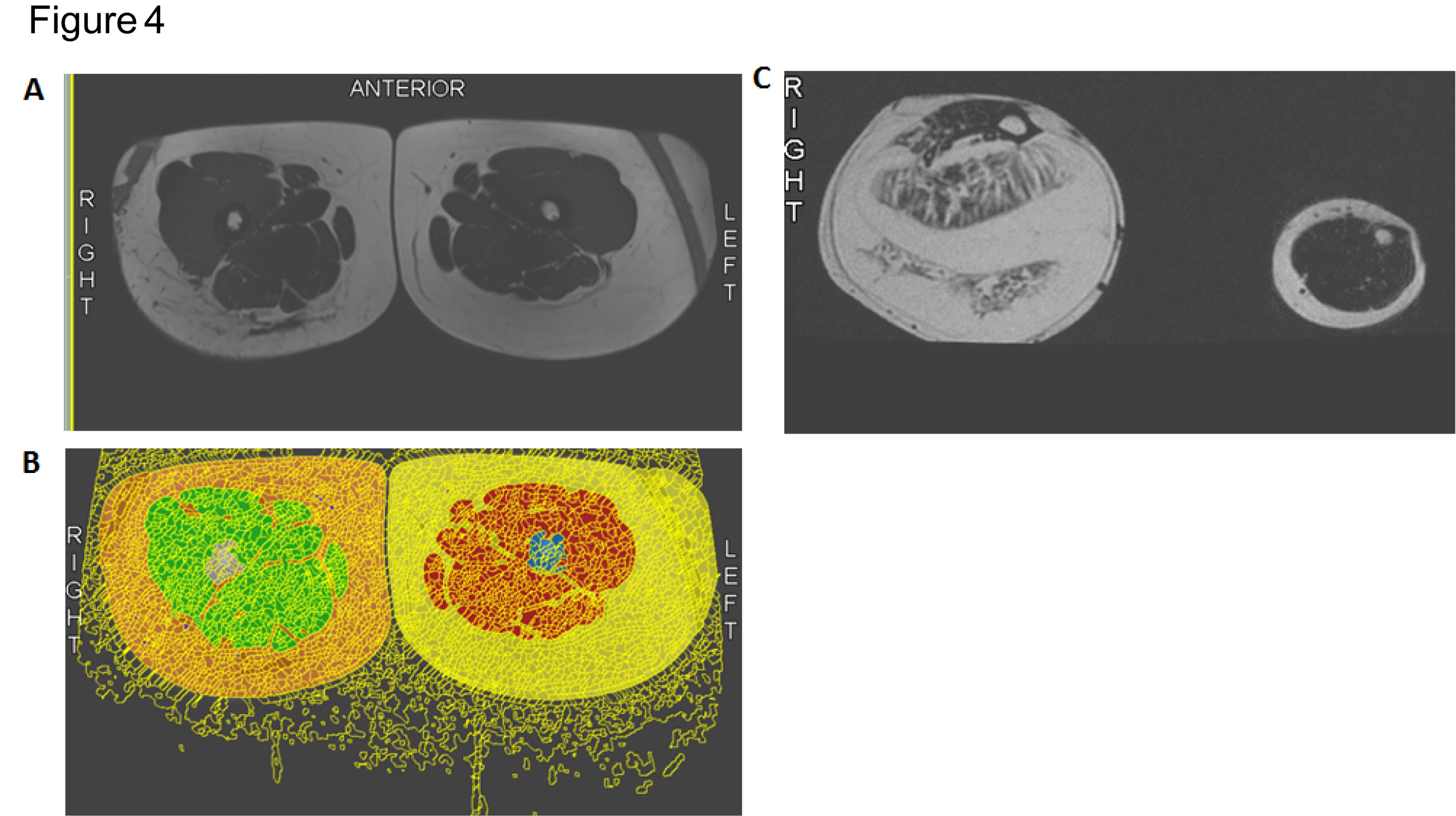


**Figure S3. Illustration of MRI analysis technique employed in study**. **A.** T1- weighted MRI images of left-sided overgrowth of the thigh in a participant. **B.** Analysis of tissue composition in the same participant using volumetric software. **C.** MRI image demonstrating marbling of fat in muscle and difficulties in differentiating tissue planes.


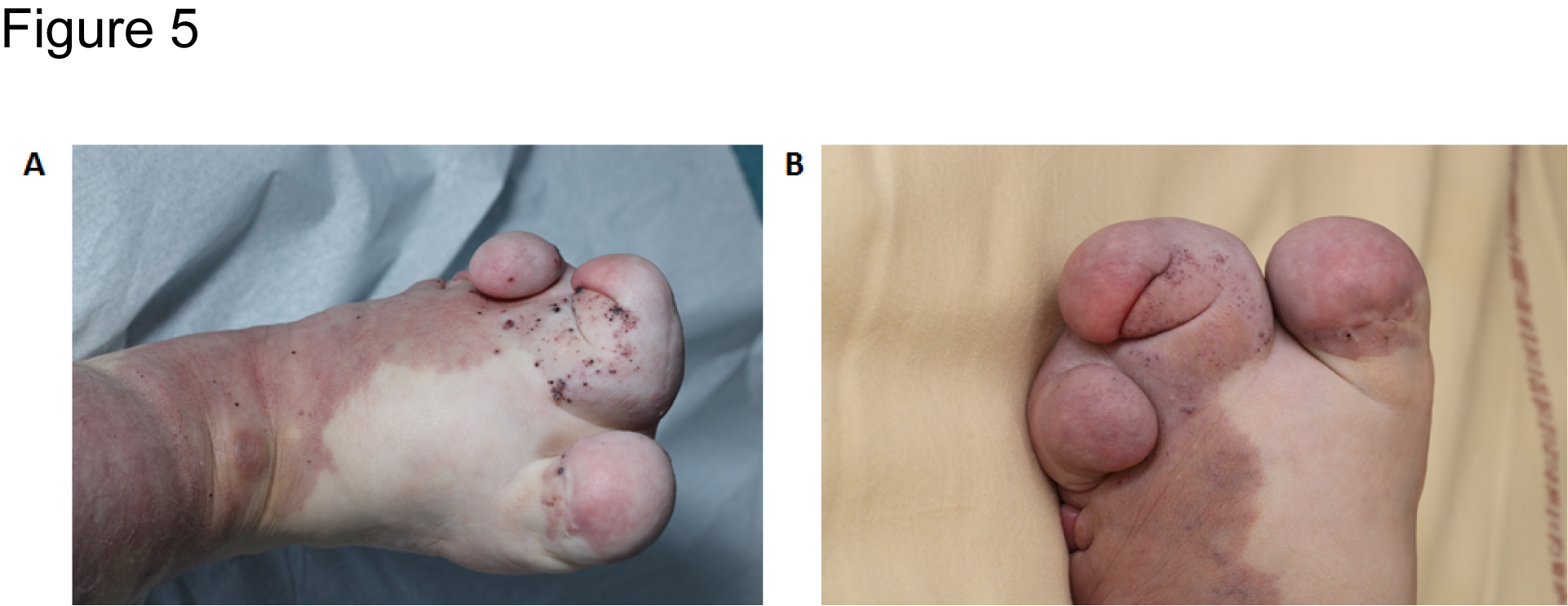


**Figure S4. Image of cutaneous lymphangiectasis before and after therapy.** Images **A.** before and **B.** after sirolimus therapy in the same participant,

demonstrating partial remission of lymphangiectasis.
